# Fuzzy Inference and Sequence Model-Based Collision Risk Prediction System for Stand-On Vessel

**DOI:** 10.3390/s22134983

**Published:** 2022-07-01

**Authors:** Ho Namgung, Sung-Wook Ohn

**Affiliations:** 1 Division of Navigation and Information System, Mokpo National Maritime University, Mokpo 58628, Korea; ngh2009@mmu.ac.kr; 2 Department of Maritime Transportation System, Mokpo National Maritime University, Mokpo 58628, Korea

**Keywords:** International Regulations for Preventing Collision at Sea, collision risk, stand-on vessels, collision risk prediction system, fuzzy inference system

## Abstract

Although the International Regulations for Preventing Collision at Sea (COLREGs) provide guidelines for determining the encounter relations between vessels and assessing collision risk, most collision accidents occur in crossing situations. Accordingly, prior studies have investigated methods to identify the relation between the give-way and stand-on vessels in crossing situations to allow the stand-on vessel to make the optimal collision-avoidance decision. However, these studies were hindered by several limitations. For example, the collision risk at the current time (*t*) was evaluated as an input variable obtained at the current time (*t*), and collision-avoidance decisions were made based on the evaluated collision risk. To address these limitations, a collision risk prediction system was developed for stand-on vessels using a fuzzy inference system based on near-collision (FIS-NC) and a sequence model to facilitate quicker collision avoidance decision making. This was achieved by predicting the future time point (*t* + *i*) collision risk index (CRI) of the stand-on vessel at the current time point (*t*) when the own-ship is determined to be the stand-on vessel in different encounter relations. According to the performance verification results, navigators who use the developed system to predict the CRI are expected to avoid collisions with greater clearance distance and time.

## 1. Introduction

According to data compiled by the Korea Maritime Safety Tribunal [1] on vessel accidents by accident type in South Korea over the past five years (2017–2021), the most common accident type was engine damage, followed by collision, safety accidents, grounding, fire and explosion, capsizing, sinking, and contact. Vessel collision accidents are of particular concern because they not only result in structural damage to the hull but also cause loss of life and property, as well as marine pollution. The main cause of collision accidents is operational negligence, such as non-compliance with general navigational principles, laws, and regulations, which accounts for approximately 95% of all accidents. Hence, to prevent these accidents, complying with the International Regulations for Preventing Collisions at Sea (COLREGs) [2] is essential. These regulations describe the appropriate collision-avoidance actions at optimal positions and times based on the analysis of a wide variety of collected data.

**Figure A1:**
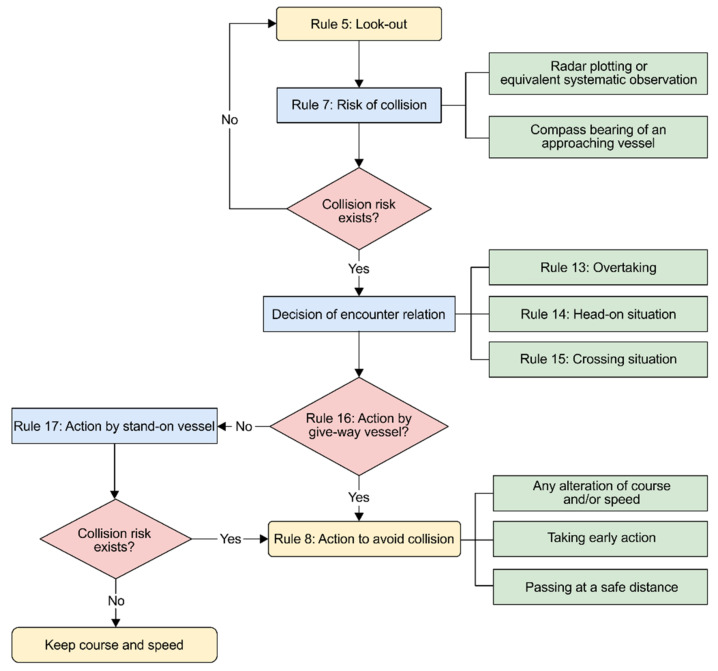
Procedure for collision avoidance based on COLREGs ([3]. 2022, Namgung, H.).

According to the navigation rules in COLREGs Part B, the collision-avoidance actions between vessels, shown in Figure A1, are performed considering Rules 5, 7, 8, and 13–17 [3]. In particular, the collision risks are assessed, and encounter relations are determined according to the following rules, as shown in Figure A1:

“Rule 7”: Perform collision risk assessment using radar plotting or equivalent systematic observation and compass bearing of an approaching vessel.

“Rules 13–15”: Determine whether it is a head-on situation, overtaking, or a crossing situation between the own-ship (OS) and target ship (TS).

“Rule 16”: If determined to be a give-way vessel, create a safe distance early by simultaneously changing both course and speed, or only course or speed, and take action to avoid collision with the TS.

“Rule 17”: If determined to be a stand-on vessel, and if there is no risk of collision due to the collision-avoidance action of the give-way vessel, then maintain current course and speed; otherwise, take a collision-avoidance action.

Despite the vessel collision risk assessment and encounter relation determination according to Rules 7 and 13–17, most collision accidents occur in crossing situations [4,5,6]. This is because, for various crossing situations, the crew often cannot determine the relation between the give-way and stand-on vessels. Moreover, although the duty of the give-way vessel is relatively lucid and simple, the stand-on vessel must perform a collision-avoidance action after observing the give-way vessel’s navigation. Nonetheless, most of these studies are designed from the give-way ship perspective only [7,8,9,10,11,12,13,14,15]. 

Hence, conflict analysis on the activation of the stand-on ship’s role in conflict elimination to improve safe conflict resolution among ships encountering one another has been conducted as follows. To unambiguously interpret the relation between the give-way and stand-on vessels in a crossing situation and to assist the stand-on vessel in making the optimal decision to avoid collision, the ship domain (SD) [16,17,18], collision risk index (CRI) [5,19,20,21,22,23], minimum distance to collision (MDTC) [24,25,26,27,28], and nonlinear velocity obstacle (NL-VO) algorithm [6,29] have been used. The SD is a generalized safe distance that must be maintained in situations where there are no TSs or obstacles. It is used as a standard to assess the collision risk of the stand-on vessel [16,17,18]. In the CRI, if the OS is determined to be a stand-on vessel, then a collision-avoidance action is taken when the defined CRI is higher than a threshold value [5,19,20,21,22,23]. The MDTC defines the minimum distance at which the stand-on vessel can avoid a collision; when the distance between the give-way and stand-on vessels is less than the MDTC, a collision-avoidance decision is made [24,25,26,27,28]. In collision avoidance using the NL-VO algorithm, the stand-on vessel estimates the navigation intention of the give-way vessel using SLoD (Stand-on Ship as Second Line of Defense) and makes a collision-avoidance decision considering the dynamic characteristics of the vessel’s behavior using the NL-VO algorithm [6,29]. However, although the aforementioned studies were performed to reduce collision accidents in crossing situations, they did not consider several important issues. First, they considered only the relations between the give-way and stand-on vessels based on the four stages of a collision situation that is outlined in a guide on collision-avoidance rules [30]. Therefore, these studies do not consider all of the encounter relations that occur between give-way and stand-on vessels in various crossing situations. Second, the collision risk at the current time (*t*) was assessed using input variables obtained at this time, and the collision-avoidance decision was made based on the evaluated collision risk level. Thus, when assessing collision risk based on the current time point, a collision-avoidance action must be performed without clearance space or time since the navigator must make a decision when the vessel has already passed the current time point (*t*).

In this study, a collision risk prediction system was developed for stand-on vessels using a sequence model to facilitate quicker collision-avoidance decision making compared to conventional approaches by predicting the future time point (*t* + *i*) CRI of the stand-on vessel at the current time point (*t*) when the OS is determined to be the stand-on vessel in various encounter relations. First, to identify the encounter relation in various crossing situations, the give-way vessel and stand-on vessel are determined according to the encounter relation determination diagram. Additionally, for each encounter angle of each stand-on vessel, continuous input and output data are collected from the point when the collision risk between the give-way and stand-on vessels occurs to the point when the stand-on vessel must perform collision-avoidance actions. Finally, an optimal collision risk prediction system was developed based on learning using the long short-term memory (LSTM), bidirectional LSTM (Bi-LSTM), and gated recurrent unit (GRU) sequence models. 

The remainder of this paper is separated into different sections. Section 2 presents the theoretical background of this study. Section 3 outlines the system development process and describes the development of the collision risk prediction system for stand-on vessels in detail. Section 4 examines the performance evaluations based on a case study of the developed system, in addition to a detailed discussion of the results. Finally, Section 5 presents the main conclusions of the study. 

## 2. Theoretical Background

### 2.1. Determining Encounter Relations and Required Avoidance Actions

**Figure 1:**
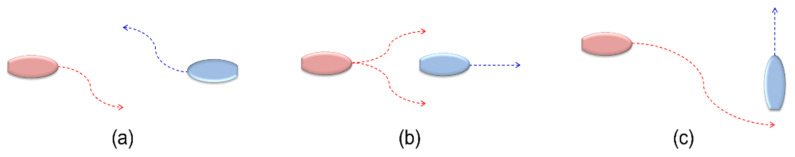
Routes of steering for collision avoidance in COLREGs.

The required avoidance actions in COLREGs rules 13–15 [2], i.e., (a) a head-on situation, (b) overtaking, and (c) a crossing situation, are schematized in Figure 1. In the head-on situation, the OS and TS have equal responsibilities for avoiding each other. In the overtaking situation, a vessel is deemed to be overtaking when approaching another vessel from a direction more than 22.5° abaft of its beam, and the overtaking vessel is not relieved of its duty to keep clear of the overtaken vessel until completely past and clear. In the crossing situation, the vessel that has the other vessel on its starboard side (i.e., the give-way vessel) must avoid the course of the other vessel and, unless circumstances permit, avoid crossing ahead of it (i.e., the stand-on vessel).

**Figure 2:**
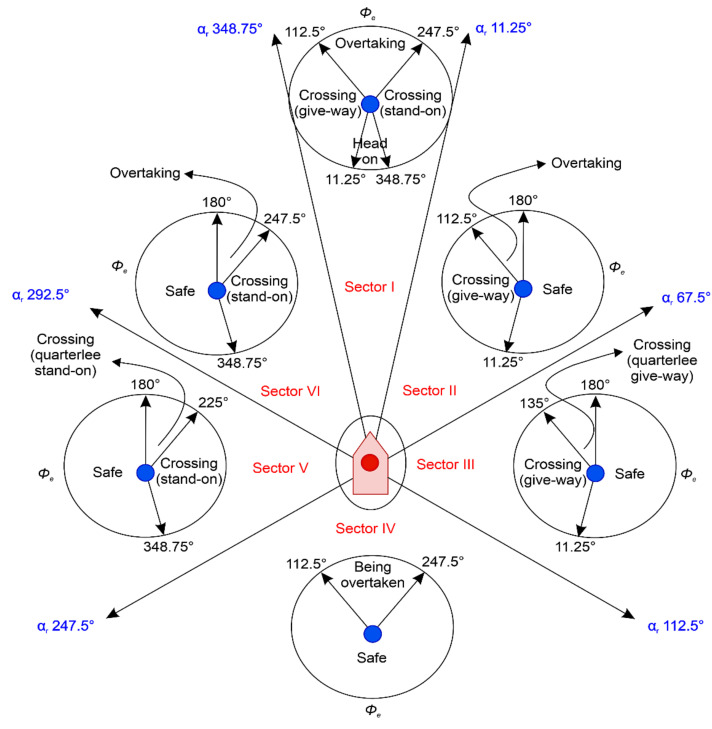
Decisions for different encounter relations ([3]. 2022, Namgung, H.).

Figure 2 is a diagram for determining the encounter relation and the required avoidance actions between the OS and TS using the visibility range of navigation lights. Based on the course $\phi_{o}$ of the OS, the position area of the TS is determined as the relative bearing $\alpha_{r}$, and the encounter angle $\phi_{e}$, where the course of the OS, $\phi_{o}$, meets that of the TS, $\phi_{t}$, in the determined area is calculated, thereby determining the encounter relation and the avoidance actions required by the OS. The circled areas indicate the TS; the OS determines the encounter relation in the circle based on $\phi_{e}$, and the OS’s avoidance actions differ depending on the encounter relation. These are classified here as head-on, crossing (give-way), crossing (quarter lee give-way), crossing (stand-on), crossing (quarter lee stand-on), overtaking, being overtaken, and safe.

**Figure 3:**
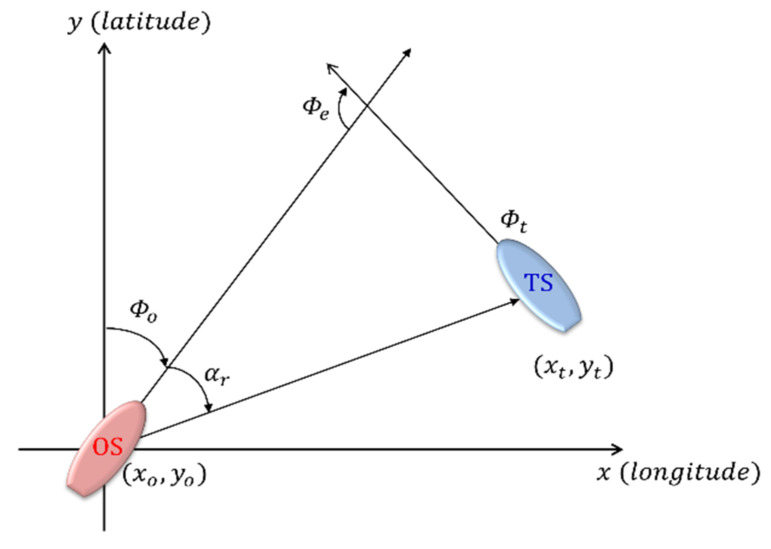
Relative bearing and encounter angle.

Here, $\alpha_{r}$ and $\phi_{e}$ between the OS and TS are geometrically related, as shown in Figure 3. Further, $\phi_{e}$ is calculated using Equation (1); if it is a negative number, then 360° is added.

(1) $$\phi_{e}=\phi_{t}-\phi_{o}-\pi$$

### 2.2. Collision Risk Inference Using FIS-NC

#### 2.2.1. Input Parameters

**Figure 4:**
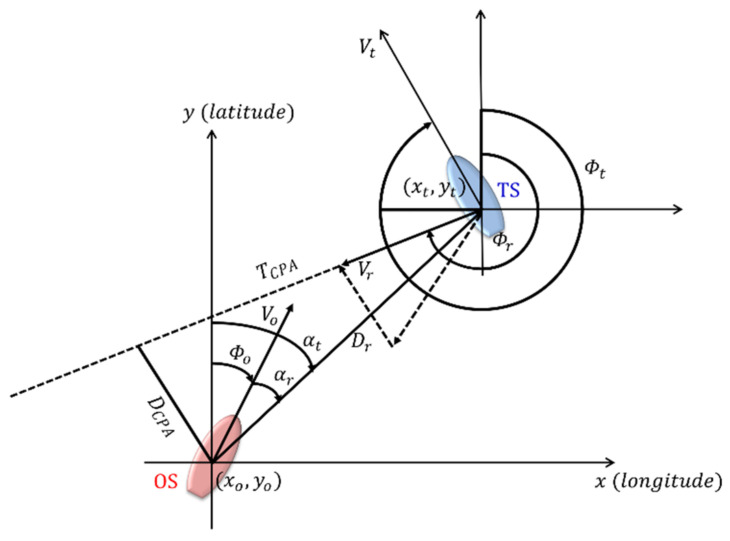
Geometry of collision of moving ships.

$D_{\mathrm{CPA}}$ is the minimum distance at which the OS passes the TS when maintaining its current course and speed in an encounter. $T_{\mathrm{CPA}}$ is the time taken to reach $D_{\mathrm{CPA}}$, the point at which the two vessels are the closest. $D_{\mathrm{CPA}}$ and $T_{\mathrm{CPA}}$ are obtained from geometric calculations, as shown in Figure 4 [3,23]. 

Here, $\left(x_{o},y_{o}\right)$, $\phi_{o}$, and $V_{o}$ represent the position, course, and speed of the OS, and $\left(x_{t},y_{t}\right)$, $\phi_{t}$, and $V_{t}$ indicate the position, course, and speed of the TS; $\alpha_{r}$ is the relative bearing of the TS with respect to the OS. The relative movement parameters, $D_{\mathrm{CPA}}$, and $T_{\mathrm{CPA}}$ are mathematically expressed using Equations (2)–(6).

(2) $$D_{r}=\sqrt{{\left(x_{t}-x_{o}\right)}^{2}+{\left(y_{t}-y_{o}\right)}^{2}}$$

(3) $$V_{r}=V_{o}\times \sqrt{1+{\left(\;\frac{V_{t}}{V_{o}}\;\right)}^{2}-2\times \frac{V_{t}}{V_{o}}\times \cos\left(\phi_{o}-\phi_{t}\right)}$$

(4) $$\phi_{r}=cos^{-1}\left(\;\frac{V_{o}-V_{t}\times \cos\left(\phi_{o}-\phi_{t}\right)}{V_{r}}\right)$$

(5) $$D_{CPA}=D_{r}\times \sin\left(\phi_{t}-\alpha_{t}-\pi \right)$$

(6) $$T_{CPA}=D_{r}\times \cos\left(\phi_{t}-\alpha_{t}-\pi \right)/V_{r}$$

where $D_{r}$ is the relative distance between the OS and TS, $V_{r}$ is the relative speed, $\phi_{r}$ is the relative course, and $\alpha_{t}$ is the true bearing of the TS. Here, the variance of compass degree (VCD) can be calculated using Equation (7).

(7) $$VCD_{i}=\left|\alpha_{r_{i}}-\alpha_{r_{i-1}}\right|$$

#### 2.2.2. CRI Inference Based on IF-THEN Rule

$D_{\mathrm{CPA}}$, $T_{\mathrm{CPA}}$, VCD, and $D_{r}$ are set to the antecedent parameter values $x_{1}$, $x_{2}$, $x_{3}$, and $x_{4}$, and CRI is set to the consequent parameter value $R_{C}$. The membership function for each fuzzy set is determined using Equation (8) based on the language variables Collision (C), Danger (D), Threat (T), and Attention (A).

$$Rule_{i}\;:\;\mathrm{If}\;x_{1}\;is\;\mu_{\left(D_{CPA}\right)i}\;\mathrm{and}\;x_{2}\;\mathrm{is}\;\mu_{\left(T_{CPA}\right)i}\;\mathrm{and}\;x_{3}\;\mathrm{is}\;\mu_{\left(VCD\right)i}\;\mathrm{and}\;x_{4}\;\mathrm{is}\;\mu_{\left(D_{r}\right)i}$$

(8) $$\;\;\;\;\;\;\;\;\;\;\;\;\;\;\;\;\;\;\;\;\;\;\;\;\;\;\;\;\;\;\;\;\;\;\;\;then\;\;R_{C}\;=f_{i}\left(x_{1},\;x_{2},\;x_{3},\;x_{4}\right)\;\;\;$$

The antecedent parameters $x_{1}$, $x_{2}$, $x_{3}$, and $x_{4}$ are expressed as $\;\mu_{\left(D_{\left(CPA\right)}\right)}$, $\mu_{\left(T_{\left(CPA\right)}\right)}$, $\;\mu_{\left(VCD\right)}$, and $\;\;\mu_{\left(D_{r}\right)}$, and the consequent parameter $R_{c}^{i}$ is the function $f_{i}\left(x_{1},\;x_{2},\;x_{3},\;x_{4}\right)$; $f_{i}\left(x_{1},\;x_{2},\;x_{3},\;x_{4}\right)$ is a polynomial expressed in Equation (9). If $k_{i,0}$, $k_{i,1}$, $k_{i,2}$, $k_{i,3}$, and $k_{i,4}$ are the consequent argument set of rule i, and $x_{1}$, $x_{2}$, $x_{3}$, and $x_{4}$ are 0, then $f_{i}$ has only the $k_{i,0}$ term.

(9) $$f_{i}=k_{i,0}\;+\;k_{i,1}\times x_{1}+k_{i,2}\times x_{2}\;+k_{i,3}\times x_{3}\;+k_{i,4}\times x_{4}\;\;\;\;\;\;\;$$

Therefore, there are a total of 256 rules in the fuzzy inference system based on near-collision (FIS-NC) that comprise combinations of the membership functions, as shown in Table 1 [23].

Given that FIS-NC has a total of 256 rules, the function $f_{i}\left(x_{1},\;x_{2},\;x_{3},\;x_{4}\right)$ can be expressed as Equation (10).

(10) $$f_{1}\left(x_{1},\;x_{2},\;x_{3},\;x_{4}\right)\;\vdots \;\;f_{256}\left(x_{1},\;x_{2},\;x_{3},\;x_{4}\right)$$

The final output expresses all 256 consequent argument sets as a single unit, which is obtained using the weighted average $\overset{⌢}{f}$, as shown in Equation (11).

(11) $$\overset{⌢}{f}\;=\sum_{i=1}^{256}\frac{\mu \left(k_{i1}\right)\times k_{i1}+\mu \left(k_{i2}\right)\times k_{i2}+\mu \left(k_{i3}\right)\times k_{i3}+\mu \left(k_{i4}\right)\times k_{i4}}{\mu \left(k_{i1}\right)+\mu \left(k_{i2}\right)+\mu \left(k_{i3}\right)+\mu \left(k_{i4}\right)}$$

The calculated CRI ranges from 0.00 to 1.00; if the time point for collision avoidance of the give-way vessel is at least 0.01 or that of the stand-on vessel is at least 0.33, then a collision-avoidance action is taken.

### 2.3. Sequence Model

If it is assumed that the give-way vessel does not perform adequate clearing action early in a crossing situation, then the stand-on vessel’s CRI gradually increases as all of the input parameters approach 0 over time. In this sequence data, since the past data influence present data, it is necessary to consider both sets of data for future prediction. A recurrent neural network (RNN) is a representative model that can be applied to sequence data to create a prediction model [31,32]. A backpropagation algorithm is used for training general RNNs. However, when error information is backpropagated to a point in the past, the gradient generally vanishes quickly. The usual approaches for mitigating this phenomenon include LSTM [33,34], Bi-LSTM [35,36,37], and GRU [38]. 

#### 2.3.1. LSTM

**Figure 5:**
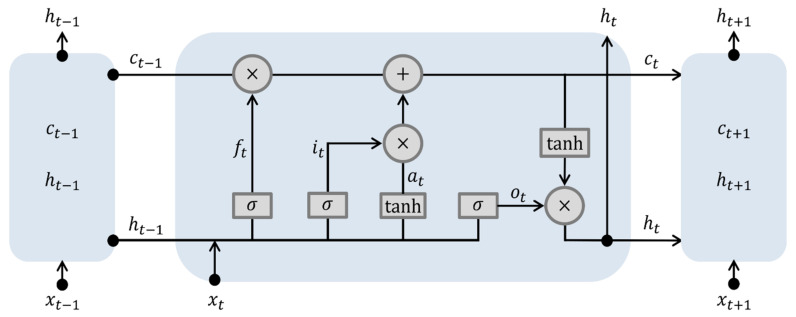
Schematic architecture of the LSTM.

In Figure 5, $x_{t}$ represents the input to the hidden node at time t, and $h_{t}$ indicates the node output at time t. The input gate $i_{t}$ plays a role in determining how much of the processing result of input information, $x_{t}$, is represented in the memory cell, $c_{t}$. The output value $i_{t}$ of the input gate is calculated using Equation (12) based on the input $x_{t}$ and its weight $U_{i}$, the output $h_{t-1}$ of the previous time point and its weight $W_{i}$, and the bias term $b_{i}$. Here, σ is the sigmoid function.

(12) $$i_{t}=\sigma \left(U_{i}x_{t}+W_{i}h_{t-1}+b_{i}\right)$$

The forget gate $f_{t}$ determines the ratio of the previous state value $c_{t-1}$ of the memory cell that should be maintained at the current time point t. The value of the forget gate is calculated using Equation (13) based on input $x_{t}$ and its weight $U_{f}$, the output $h_{t-1}$ of the previous time point and its weight $W_{f}$, and the bias term $b_{f}$.

(13) $$f_{t}=\sigma \left(U_{f}x_{t}+W_{f}h_{t-1}+b_{f}\right)$$

The value $c_{t}$ stored in the memory cell at time t is calculated using Equation (14); $a_{t}$ is the newly determined state value that is calculated using the LSTM model at time t.

(14) $$a_{t}=\tanh\left(U_{c}x_{t}+W_{c}h_{t-1}+b_{c}\right)\;c_{t}=i_{t}\circ a_{t}+f_{t}\circ c_{t-1}$$

where ∘ indicates the product of the corresponding position elements of two vectors. According to the second equation, the value $c_{t}$ of the memory cell is determined by considering the ratio, $f_{t},$ of the previous state value, $c_{t-1},$ and the ratio $i_{t}$ of the new state value $a_{t}$. The output gate $o_{t}$ plays a role in adjusting the output of the value stored in memory cell $c_{t}$. Here, $U_{o}$, $W_{o}$, and $V_{o}$ are the weights of the input, previous output, and previous state value, respectively, and $b_{o}$ is the bias term.

(15) $$o_{t}=\sigma \left(U_{o}x_{t}+W_{o}h_{t-1}+V_{o}c_{t-1}+b_{o}\right)$$

Output $h_{t}$ at time point t is calculated by multiplying the output gate values $o_{t}$ and $\tanh\left(c_{t}\right)$ for each element, as shown in Equation (16).

(16) $$h_{t}=o_{t}\circ \tanh\left(c_{t}\right)$$

Algorithm 1 state the execution of LSTM [33,34].

**Algorithm 1:** Algorithm for execution of LSTMInput: sequence data $\left(x_{1},\;x_{2},\;\cdots ,\;x_{t}\right)$weight $W_{i},\;U_{i},W_{f},U_{f},\;\;W_{o},\;U_{c},\;W_{c}\;U_{o},\;V_{o}$bias $b_{i},\;b_{f},\;b_{c},\;b_{o}$output: $h_{1},\;h_{2},\cdots ,\;h_{t}$ 1 $h_{0}\leftarrow 0$2 $c_{0}\leftarrow 0$3      **for**
*t* = 1 to *t*4           $i_{t}\leftarrow \sigma \left(U_{i}x_{t}+W_{i}h_{t-1}+b_{i}\right)$5           $a_{t}\leftarrow \tanh\left(U_{c}x_{t}+W_{c}h_{t-1}+b_{c}\right)$6           $f_{t}\leftarrow \sigma \left(U_{f}x_{t}+W_{f}h_{t-1}+b_{f}\right)$7           $c_{t}\leftarrow i_{t}\circ a_{t}+f_{t}\circ c_{t-1}$8           $o_{t}\leftarrow \sigma \left(U_{o}x_{t}+W_{o}h_{t-1}+V_{o}c_{t-1}+b_{o}\right)$9           $h_{t}\leftarrow o_{t}\circ \tanh\left(c_{t}\right)$10     **end**

#### 2.3.2. Bi-LSTM

**Figure 6:**
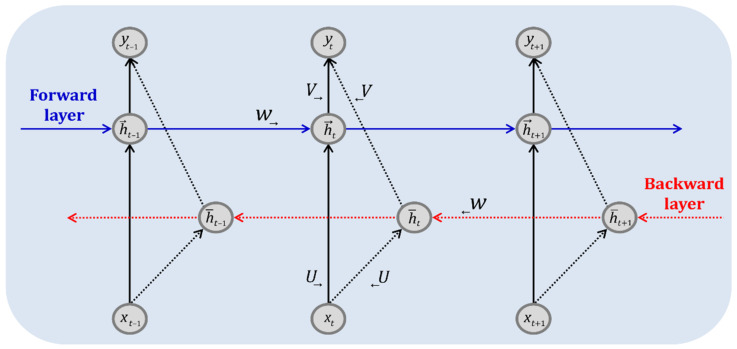
Schematic architecture of the Bi-RNN.

Bi-RNN is a model in which the output at time point t is affected by not only the input and hidden layer values at a previous time but also the input and hidden layer values at a later time. Figure 6 shows the architecture of a Bi-RNN, in which the output is obtained from two hidden layer nodes. The connected lines from left to right indicate that the past has an influence. In contrast, the connected lines from right to left indicate that the future influences the present. Here, Bi-LSTM uses an LSTM model for each hidden layer of the former.

If we define the weight matrix between the input and forward layers as $U_{\to }$, the weight matrix between the forward layers as $W_{\to }$, and the bias term vector of the forward layer as $b_{\to }$, then the forward layer value $\vec{h_{t}}$ at time t can be calculated using Equation (17).

(17) $$\vec{h_{t}}=\sigma \left(U_{\to }x_{t}+W_{\to }{\vec{h}}_{t-1}+b_{\to }\right)$$

If we define the weight matrix between the input and backward layers as $U_{\leftarrow }$, the weight matrix between the backward layers as $W_{\leftarrow }$, and the bias term vector of the backward layer as $b_{\leftarrow }$, then the backward layer value ${\bar{h}}_{t}$ at time t can be calculated as in Equation (18).

(18) $${\bar{h}}_{t}=\sigma \left(U_{\leftarrow }x_{t}+W_{\leftarrow }{\bar{h}}_{t+1}+b_{\leftarrow }\right)$$

The output layer value $y_{t}$ at time t is calculated as shown in Equation (19) by combining the forward layer value $\vec{h_{t}}$ and backward layer value ${\bar{h}}_{t}$. $V_{\to }$ is the weight matrix between the forward and output layers, $V_{\leftarrow }$ is the weight matrix between the backward and output layers, $b_{o}$ is the bias term, and f is the activation function.

(19) $$y_{t}=f\left(V_{\to }{\vec{h}}_{t}+V_{\leftarrow }{\bar{h}}_{t}+b_{o}\right)$$

#### 2.3.3. GRU

**Figure 7:**
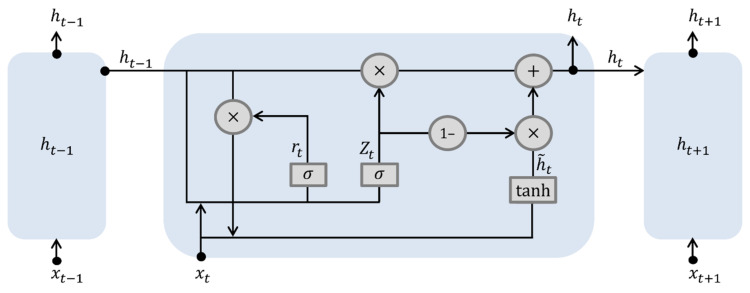
Schematic architecture of the GRU.

Like LSTM in RNN, the GRU model is proposed to solve the vanishing gradient problem, and its internal operation is simpler than that of the LSTM. Figure 7 shows the architecture of the GRU model. While LSTM has three gates, GRU has only two gates: reset and update. The reset gate r decides how to combine input $x_{t}$ and existing stored content $h_{t-1}$. The reset gate performs computations according to Equation (20).

(20) $$r_{t}=\sigma \left(U_{r}x_{t}+W_{r}h_{t-1}+b_{r}\right)$$

Equation (21) can be used to calculate a new internal state value ${\tilde{h}}_{t}$ by combining input $x_{t}$ and the existing stored content $h_{t-1}$ according to the reset gate value $r_{t}$.

(21) $${\tilde{h}}_{t}=\tanh(U_{h}x_{t}+W_{h}\left(r_{t}\circ h_{t-1}\right)$$

The update gate z determines the ratio that represents the existing stored value $h_{t-1}$ and newly calculated value ${\tilde{h}}_{t}$. The value of the update gate $z_{t}$ is calculated using $h_{t-1}$ and input $x_{t}$ according to Equation (22).

(22) $$z_{t}=\sigma \left(U_{z}x_{t}+W_{z}h_{t-1}+b_{z}\right)$$

The internal state value, $h_{t},$ is calculated by combining the existing stored content $h_{t-1}$ and newly calculated internal state value ${\tilde{h}}_{t}$ according to the updated gate value obtained from Equation (23).

(23) $$h_{t}=z_{t}\circ h_{t-1}+\left(1-z_{t}\right)\circ {\tilde{h}}_{t}$$

The internal state value, $h_{t},$ is the output value without modification. Therefore, in the GRU model, the internal state value and output data have the same dimensions.

## 3. Stand-On Vessel Collision Risk Prediction System Using FIS-NC and Sequence Model

### 3.1. System Development Process

**Figure 8:**
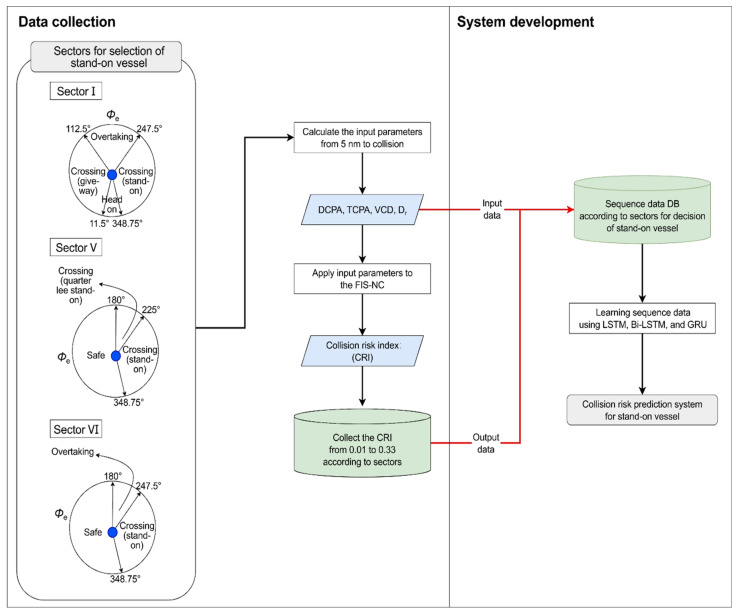
Development process of collision risk prediction system for stand-on vessel.

As shown in Figure 8, the development process of the stand-on vessel collision risk prediction system is divided into data collection and system development. In data collection, sectors I, V, and VI in the encounter relation determination diagram of Figure 2 are established using $\alpha_{r}$, and in the determined sectors, $\phi_{e}$ is used to judge whether the vessel is a stand-on vessel. Second, if the vessel is judged to be a stand-on vessel, then according to the guide on collision avoidance rules [30], the input parameters $D_{\mathrm{CPA}}$, $T_{\mathrm{CPA}}$, VCD, and $D_{r}$ are calculated from 5 nautical miles (nm), the boundary point where the give-way vessel and stand-on vessel lose freedom of movement, to the point where both vessels collide. Third, the calculated parameters are input to FIS-NC to infer the CRI. Fourth, the CRIs from 0.01 (time point for collision avoidance of the give-way vessel) to 0.33 (time point for collision avoidance of the stand-on vessel) that are inferred based on the FIS-NC and input parameters used for this inference are collected. During system development, the collected input parameters and CRIs from 0.01 to 0.33 are designated as the input and target data of the sequence data, respectively. Additionally, after the values are input to LSTM, Bi-LSTM, and GRU and learned to develop a stand-on vessel collision risk prediction system for each case, their performances are compared. 

### 3.2. Data Collection

#### 3.2.1. Simulation Scenario

After configuring the scenarios shown in Table 2, data were collected via simulation using MATLAB. First, we set the speed of both vessels to 15 kn, the highest speed limit in Korean ports [39]. Second, the automatic identification system (AIS) dynamic information was updated according to the required changes in the vessel’s speed and course to prevent a collision between the vessels. Since the reporting period was 4 s when the speed was set to 15 kn, the AIS period was set to 4 s [40]. Third, the $D_{r}$ was calculated considering the relative speed of both vessels and an AIS period of 4 s, according to the guide on collision-avoidance rules [30], from 5 nm, the boundary point where the give-way and stand-on vessels lose freedom of movement, until collision. Finally, in sectors I, V, and VI, the OS’s heading was set to 000 degrees as the reference, and the TS’s heading was set as 11.25° to allow the OS to become the stand-on vessel for the circles in sectors I, V, and VI. Here, the $\alpha_{r}$ of the TS for each OS reference was set to 360° in sector I, 270° in sector V, and 320.625° in sector VI. 

#### 3.2.2. Collected Data

Based on the simulation results for each scenario, the input parameters $D_{\mathrm{CPA}}$, $T_{\mathrm{CPA}}$, VCD, and $D_{r}$ and the CRIs that were inferred by applying the parameters in FIS-NC were collected from sectors I and VI (1680 data) and sector V (2520 data). Table 3 shows the averages of the $D_{\mathrm{CPA}}$, $T_{\mathrm{CPA}}$, VCD, $D_{r}$, and CRI data collected from sectors I, V, and VI from CRI 0.01 (the time point for collision avoidance of the give-way vessel) to 0.33 (the time point for collision avoidance of the stand-on vessel). These values represent the characteristics of the sequence model for learning data according to the flow of time. As a result, 40, 39, and 40 data values were acquired in sectors I, V, and VI, respectively.

### 3.3. System Development

**Figure 9:**
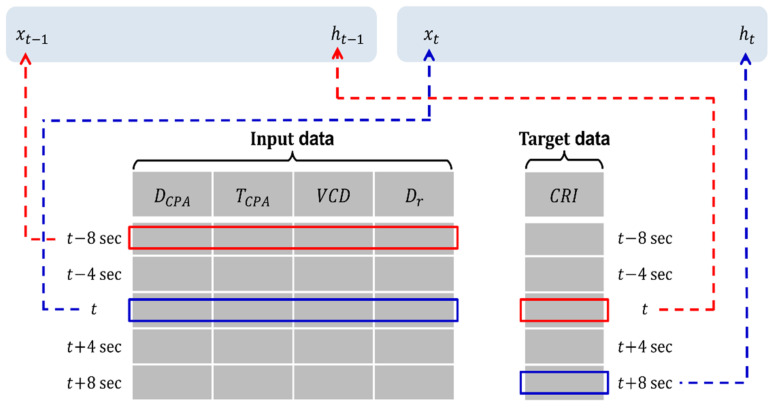
Schematic of the sequence model used for learning data.

**Figure 10:**
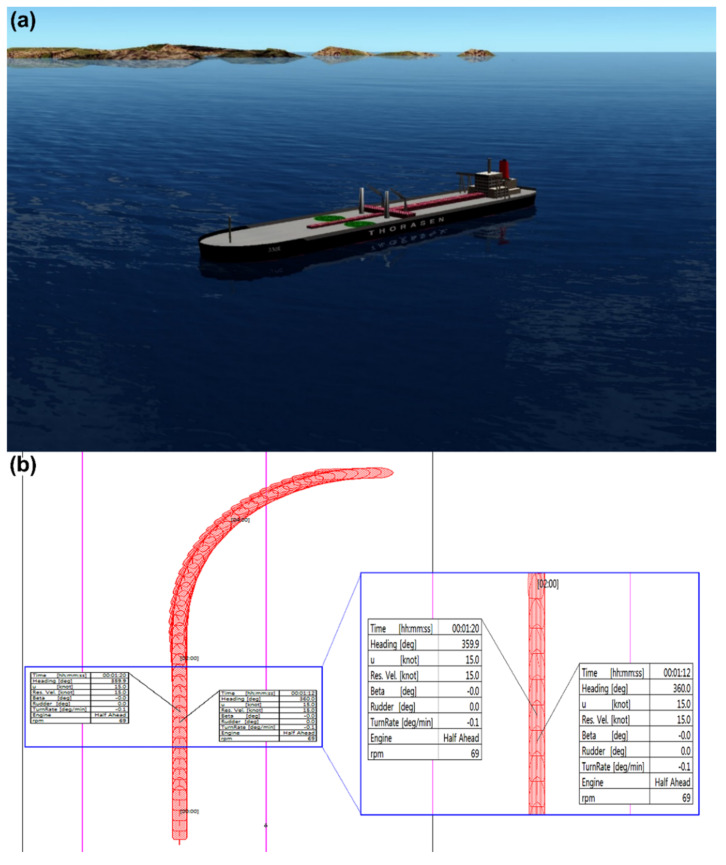
Ship handling simulator: (**a**) tanker ship and (**b**) measured trajectory.

$D_{\mathrm{CPA}}$, $T_{\mathrm{CPA}}$, VCD, and $D_{r}$, which are the input data obtained via data collection, and the CRI, which is the output data, were input to LSTM, Bi-LSTM, and GRU for learning. To predict the CRI for a certain time step in the future, the target data were determined using data that were moved twice at each time step of the input data, as shown in Figure 9. Among all vessels, the ship with the slowest reaction time is the tanker. Therefore, the difference in time was applied using a tanker. In the ship handling simulator shown in Figure 10, the laden 330,000-tonnage tanker ship experienced a kick phenomenon from its original course. The time point of this incidence was taken as the reaction time, which was measured at 8 *s* via the ship handling simulator in Figure 10.

To compare the systems developed using LSTM, Bi-LSTM, and GRU, each neural network was configured with the same conditions (hidden nodes of identical layers, number of learning epochs, gradient threshold, and initial learning rate). Adaptive moment estimation (ADMA), a method for learning weights while adjusting the learning rate for each weight, was utilized during learning. The root-mean-squared error (RMSE) was used as the selection criterion for each prediction system. RMSE is a measure of prediction error and was used to show the difference between the CRI (${\hat{R}}_{c_{i}}$) predicted using the test data of the explanatory variable in the system trained with the training data and the CRI ($R_{c_{i}}$) contained in the test data of the response variable. It can be calculated as shown in Equation (24).

(24) $$RMSE\;=\;\sqrt{\;\frac{\sum_{i=1}^{n}{\left({\hat{R}}_{c_{i}}-R_{c_{i}}\right)}^{2}}{n}}$$

**Figure 11:**
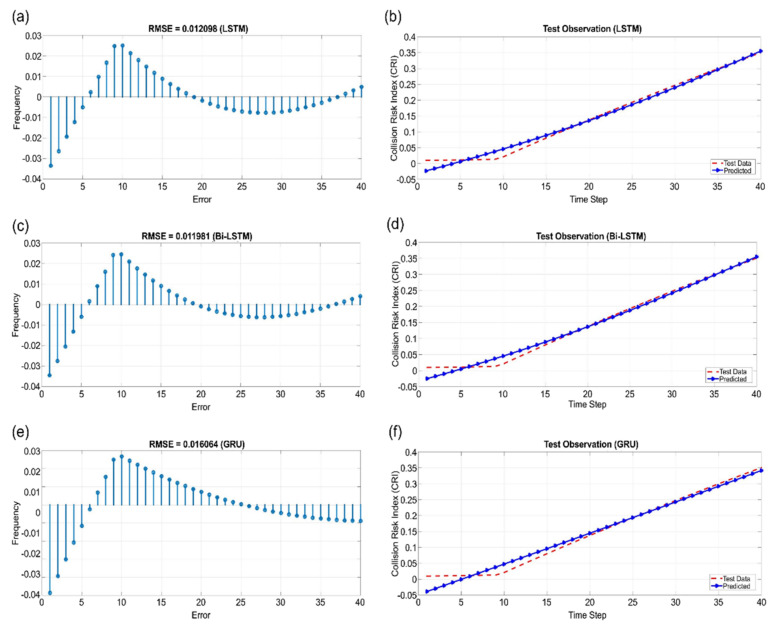
Sector I: (**a**) RMSE of LSTM, (**b**) test observation of LSTM, (**c**) RMSE of Bi-LSTM, (**d**) test observation of Bi-LSTM, (**e**) RMSE of GRU, and (**f**) test observation of GRU.

**Figure 12:**
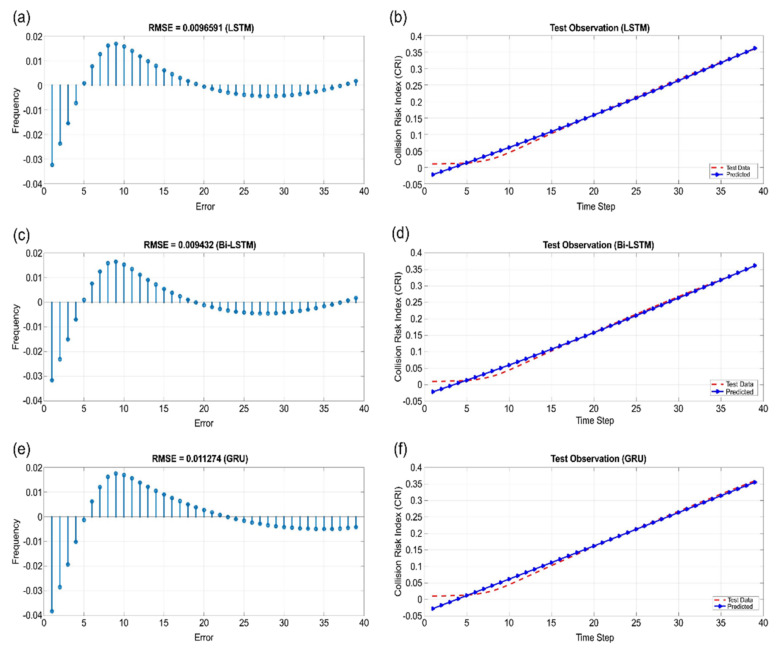
Sector V: (**a**) RMSE of LSTM, (**b**) test observation of LSTM, (**c**) RMSE of Bi-LSTM, (**d**) test observation of Bi-LSTM, (**e**) RMSE of GRU, and (**f**) test observation of GRU.

**Figure 13:**
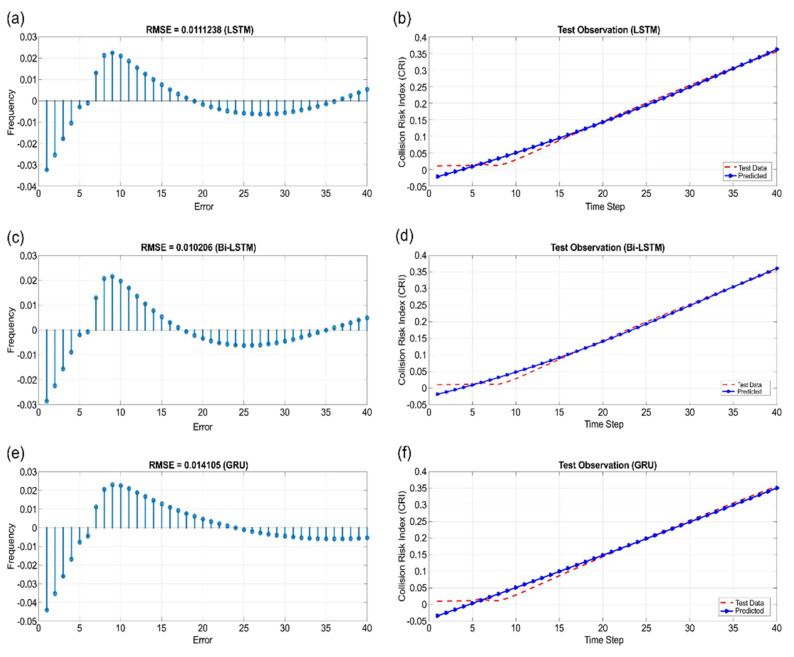
Sector VI: (**a**) RMSE of LSTM, (**b**) test observation of LSTM, (**c**) RMSE of Bi-LSTM, (**d**) test observation of Bi-LSTM, (**e**) RMSE of GRU, and (**f**) test observation of GRU.

Figure 11, Figure 12 and Figure 13 show the learning results of sectors I, V, and VI for the test data predicted using LSTM, Bi-LSTM, and GRU. According to the results, the sequence model with RMSE values that were closest to 0 in all sectors was Bi-LSTM.

### 3.4. System Application

The collision risk prediction system for stand-on vessels that was developed based on Bi-LSTM can be implemented using the following Algorithm 2. Initially, when the CRI is less than 0.01, the OS maintains its original course and speed. However, when the CRI is 0.01 or greater, the OS determines whether the TS is located in sectors I, V, or VI depending on $\alpha_{r}$. In each sector, $\phi_{e}$ is then used to determine whether the OS is a stand-on vessel; if so, then the collision-avoidance time point of the stand-on vessel, CRI= 0.33, is predicted using the developed system.
**Algorithm 2:** Algorithm for developed system applicationInput: $\alpha_{r}$, $\phi_{e}$, encounter relation $E_{r}$, $D_{CPA}$, $T_{CPA}$, VCD, $D_{r}$output: CRI1 Initialize CRI < 0.01 ← keep course and speed2    **while**
CRI
*>=* 0.01 **do**3        **if** TS is in “sector I, V, and VI” decided with $\alpha_{r}$4            **if** ($\phi_{e}$ > 247.5) and (348.75 > $\phi_{e}$) or ($\phi_{e}$ > 180) and (348.75 > $\phi_{e}$) or ($\phi_{e}$ > 247.5) and (348.75 > $\phi_{e}$) **then**5          decide $E_{r}$ ← crossing situation (stand-on, quarterlee stand-on) of OS6          predict *CRI* 0.33 ← the time point for collision avoidance of stand-on vessel7            **else**8          decide $E_{r}$ ← head-on situation or overtaking or Safe of OS9         **end**10     **end**11 **end**

## 4. Case Study

**Figure 14:**
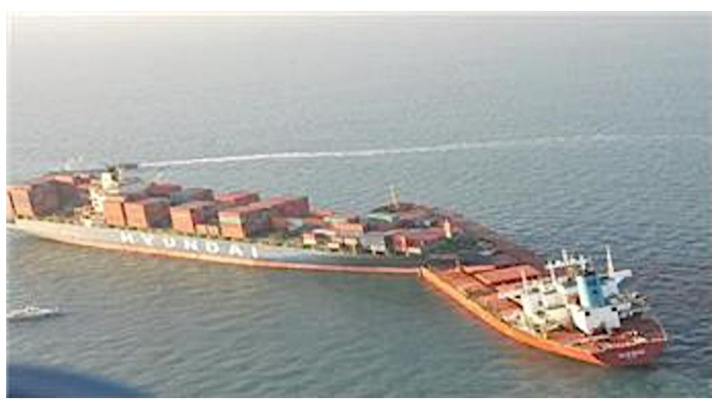
Collision accident between HYUNDAI CONFIDENCE and PACIFIC CARRIER.

According to the Korea Maritime Safety Tribunal decision [1], on 14 December 2011 at approximately 06:24 at 34° 33′ 17″ N, 128° 01′ 49″ E, PACIFIC CARRIER (stand-on vessel), which was carrying 133,104 t of coal to Samcheonpo Port, collided with HYUNDAI CONFIDENCE (give-way vessel), which was carrying 3133 twenty-foot equivalent units (TEUs) of containers from Gwangyang Port to Busan Port. The two vessels encountered a crossing situation wherein HYUNDAI CONFIDENCE was unable to avoid PACIFIC CARRIER, and the two vessels collided, as shown in Figure 14. Consequently, PACIFIC CARRIER experienced a puncture in the hull approximately 20 m long and 10 m high at the center of the left side, severe damage to cargo holds 4, 5, and 6, and approximately 70 cm of flooding in the engine room. HYUNDAI CONFIDENCE experienced severe damage to the bow and cargo hold 1. In this study, we determined whether it was possible to perform a collision-avoidance action with sufficient space and time clearance by applying the developed system to PACIFIC CARRIER and determining the encounter relation between the give-way and stand-on vessels in a crossing situation.

### 4.1. Simulation Results

**Figure 15:**
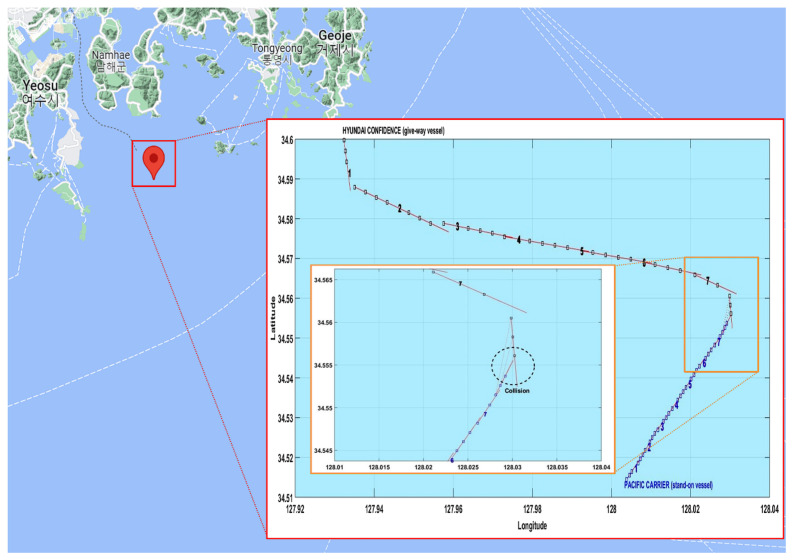
Ship trajectories of the collision accident between HYUNDAI CONFIDENCE and PACIFIC CARRIER.

Table 4 shows the trajectory data of both vessels based on the AIS information. The trajectory data were interpolated in 30 s increments and simulated using MATLAB, as shown in Figure 15. The trajectory numbers were set to simultaneously identify the movements of both vessels. Based on the analysis of trajectories from the AIS information of both vessels, trajectory number 1 had a $D_{r}$ of 6.4 nm and a relative speed of 27 kn and showed no encounter relation. Given trajectory number 2, however, with a $D_{r}$ of 5.5 nm and a relative speed of 21.2 kn, based on PACIFIC CARRIER (stand-on vessel), the encounter relation in sector VI of Figure 15 was initiated, and neither vessel took appropriate collision-avoidance actions, which led to the collision accident.

**Figure 16:**
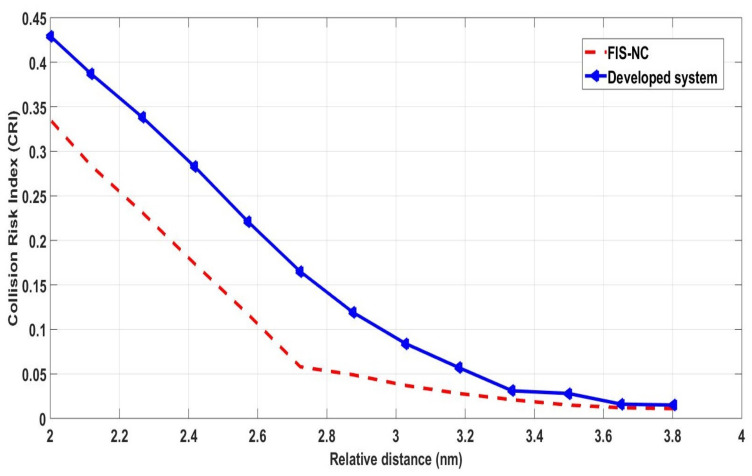
Comparison of results for CRI.

To evaluate performance, FIS-NC and the developed system were applied to PACIFIC CARRIER (stand-on vessel), and the results are shown in Figure 16 and Table 5. For FIS-NC and the developed system, in trajectory number 3, the CRI gradually increased according to the input variable from 0.01 and higher (time point for collision avoidance of the give-way vessel). In trajectory number 4, however, the developed system gave a greater $D_{r}$ and $T_{\mathrm{CPA}}$ clearance of 0.297 nm and 1 min, respectively, compared to FIS-NC at CRI = 0.33 and higher (time point for collision avoidance of the stand-on vessel) and recommended a collision-avoidance action for PACIFIC CARRIER (stand-on vessel).

### 4.2. Discussion

By applying the Algorithm 2 for the developed system to PACIFIC CARRIER (stand-on vessel) in a real collision accident, it was not only possible to accurately determine whether the vessel was the stand-on vessel in a changing encounter relation, but a collision-avoidance action was also recommended for PACIFIC CARRIER (stand-on vessel) for CRI values of 0.33 and higher (time point for collision avoidance of the stand-on vessel) assuming that HYUNDAI CONFIDENCE (give-way vessel) did not perform an appropriate collision-avoidance action. However, FIS-NC also recommended the same collision-avoidance action for PACIFIC CARRIER (stand-on vessel) from CRI 0.33 and higher (time point for collision avoidance of the stand-on vessel). 

Because each system infers and predicts different CRIs according to changes in the input variables at the same distance and time, the clearance distance and time for PACIFIC CARRIER (stand-on vessel) to avoid collision were different. To evaluate whether the avoidance actions recommended at the clearance distance and time for the CRIs inferred and predicted using each system were appropriate, the results were analyzed based on a guide for collision avoidance rules [30]. Accordingly, if the give-way vessel does not take appropriate action in the open sea according to Rule 16, the stand-on vessel must take a collision-avoidance action within 6 min at approximately 2 to 3 nm. We compared $D_{r}$ and $T_{\mathrm{CPA}}$ of FIS-NC and the developed system when the inferred and predicted CRIs were 0.33 and higher, as shown in Table 6. The developed system recommended collision-avoidance actions that satisfied the distance and time requirements in the guide to collision avoidance rules. Hence, navigators who use the system to predict CRI are expected to be able to safely avoid collisions while securing more clearance distance and time.

## 5. Conclusions

In this study, a collision risk prediction system was developed using FIS-NC and a sequence model to enable stand-on vessels to perform safe collision-avoidance actions while securing appropriate clearance distance and time. This was achieved by predicting the future CRI if the OS was determined to be a stand-on vessel in various encounter relations. The development of the collision risk prediction system for stand-on vessels was divided into data collection and system development. In data collection, when the OS was determined to be the stand-on vessel according to the encounter relation judgment guidelines, CRI values from 0.01 (time point for collision avoidance of the give-way vessel) to 0.33 (time point for collision avoidance of the stand-on vessel) that were inferred using FIS-NC and the input parameters $D_{\mathrm{CPA}}$, $T_{\mathrm{CPA}}$, VCD, and $D_{r}$ used for this inference were collected. In system development, the collected input parameters and CRIs from 0.01 to 0.33 were designated as the input data and target data of the sequence data, respectively. Additionally, after the values were input to the sequence models (LSTM, Bi-LSTM, and GRU) and learned to develop a stand-on vessel collision risk prediction system for each case, the optimal system was selected. According to the results, the collision risk prediction system for collision avoidance of stand-on vessels using Bi-LSTM demonstrated superior performance. Among vessels that experienced collision accidents, the developed system using Bi-LSTM was applied to a stand-on vessel to evaluate its performance. In this instance, the system recommended a collision-avoidance action that could be safely performed by the stand-on vessel while also securing more clearance distance and time compared to conventional approaches. However, despite the diverse maritime navigation environments and vessel navigation information encountered during voyages, only the input parameters $D_{\mathrm{CPA}}$, $T_{\mathrm{CPA}}$, VCD, and $D_{r}$ are required for the proposed system. Therefore, in future studies, systems that can predict the optimal CRI by considering additional navigation environmental factors should be investigated.

## Figures and Tables

**Table 1:** Components of the fuzzy inference rules for FIS-NC.

Rulei	$D_{CPA}$	$T_{CPA}$	VCD	$D_{r}$
Rule 1	Danger	Danger	Danger	Danger
Rule 2	Threat	Threat	Threat	Threat
Rule 3	Attention	Attention	Attention	Attention
⋮	⋮	⋮	⋮	⋮
Rule 254	Collision	Threat	Danger	Collision
Rule 255	Collision	Threat	Danger	Danger
Rule 256	Danger	Danger	Threat	Threat

**Table 2:** Scenario for simulation between OS and TS.

Sector I		Sector V		Sector VI	
OS (Stand-On Vessel)	TS (Give-Way Vessel)	OS (Stand-On Vessel)	TS (Give-Way Vessel)	OS (Stand-On Vessel)	TS (Give-Way Vessel)
000°	168.75°	000°	168.75°	000°	168.75°
000°	157.5°	000°	157.5°	000°	157.5°
000°	146.25°	000°	146.25°	000°	146.25°
000°	135°	000°	135°	000°	135°
000°	123.75°	000°	123.75°	000°	123.75°
000°	112.5°	000°	112.5°	000°	112.5°
000°	101.25°	000°	101.25°	000°	101.25°
000°	090°	000°	090°	000°	090°
000°	078.75°	000°	078.75°	000°	078.75°
000°	067.5°	000°	067.5°	000°	067.5°
-	-	-	056.25°	-	-
-	-	-	045°	-	-
-	-	-	033.75°	-	-
-	-	-	022.5°	-	-
-	-	-	011.25°	-	-

**Table 3:** Collected data in sector I.

Sector I									
$D_{\mathrm{CPA}}\;(\mathrm{nm})$	$T_{\mathrm{CPA}}\;(\min)$	VCD(degree)	$D_{r}\;(\mathrm{nm})$	CRI	$D_{\mathrm{CPA}}\;(\mathrm{nm})$	$T_{\mathrm{CPA}}\;(\min)$	VCD(degree)	$D_{r}\;(\mathrm{nm})$	CRI
0.086661	6.996518	0	3.11	0.01089	0.069942	5.646707	0	2.51	0.126185
0.085825	6.929028	0	3.08	0.01107	0.069106	5.579217	0	2.48	0.137516
0.084989	6.861537	0	3.05	0.01113	0.06827	5.511726	0	2.45	0.148778
0.084153	6.794046	0	3.02	0.01118	0.067434	5.444236	0	2.42	0.159971
0.083317	6.726556	0	2.99	0.01125	0.066598	5.376745	0	2.39	0.171095
0.082481	6.659065	0	2.96	0.01157	0.065762	5.309255	0	2.36	0.182153
0.081645	6.591575	0	2.93	0.01189	0.064926	5.241764	0	2.33	0.193143
0.080809	6.524084	0	2.9	0.01208	0.06409	5.174274	0	2.3	0.204067
0.079973	6.456594	0	2.87	0.01265	0.063254	5.106783	0	2.27	0.214926
0.079137	6.389103	0	2.84	0.01297	0.062418	5.039293	0	2.24	0.22572
0.078301	6.321613	0	2.81	0.01302	0.061582	4.971802	0	2.21	0.236449
0.077465	6.254122	0	2.78	0.020979	0.060746	4.904312	0	2.18	0.247114
0.076629	6.186632	0	2.75	0.032964	0.05991	4.836821	0	2.15	0.257716
0.075794	6.119141	0	2.72	0.044873	0.059074	4.769331	0	2.12	0.268255
0.074958	6.051651	0	2.69	0.056708	0.058238	4.70184	0	2.09	0.278732
0.074122	5.98416	0	2.66	0.068468	0.057402	4.63435	0	2.06	0.289148
0.073286	5.91667	0	2.63	0.080156	0.056566	4.566859	0	2.03	0.299502
0.07245	5.849179	0	2.6	0.09177	0.055731	4.499369	0	2	0.309796
0.071614	5.781689	0	2.57	0.103313	0.054895	4.431878	0	1.97	0.32003
0.070778	5.714198	0	2.54	0.114785	0.054059	4.364387	0	1.94	0.330205

**Table 4:** AIS trajectory data between HYUNDAI CONFIDENCE and PACIFIC CARRIER.

Trajectory Number	$D_{r}$ (nm)	$V_{r}$ (kn)	HYUNDAI CONFIDENCE (Give-Way Vessel)		PACIFIC CARRIER (Stand-On Vessel)	
			Heading (degree)	Speed (kn)	Heading (degree)	Speed (kn)
1	6.4	27.0	174	19.8	028	8.2
	6.2	27.0	174	19.8	028	8.2
	6.0	27.0	174	19.8	028	8.2
	5.9	27.0	174	19.8	028	8.2
	5.7	27.0	174	19.8	028	8.2
2	5.5	21.2	120	18.8	025	8.4
	5.3	21.2	120	18.8	025	8.4
	5.1	21.2	120	18.8	025	8.4
	4.9	21.2	120	18.8	025	8.4
	4.8	21.2	120	18.8	025	8.4
	4.6	21.2	120	18.8	025	8.4
	4.4	21.2	120	18.8	025	8.4
	4.2	21.2	120	18.8	025	8.4
3	4.1	18.5	104	18.8	029	8.5
	4.0	18.5	104	18.8	029	8.5
	3.8	18.5	104	18.8	029	8.5
	3.7	18.5	104	18.8	029	8.5
	3.5	18.5	104	18.8	029	8.5
4	3.3	18.9	102	19.4	028	8.7
	3.2	18.9	102	19.4	028	8.7
	3.0	18.9	102	19.4	028	8.7
	2.9	18.9	102	19.4	028	8.7
	2.7	18.9	102	19.4	028	8.7
	2.6	18.9	102	19.4	028	8.7
	2.4	19.0	102	19.4	028	8.8
	2.3	19.0	102	19.4	028	8.8
	2.1	19.0	102	19.4	028	8.8
	2.0	19.0	102	19.4	028	8.8
	1.8	19.0	102	19.4	028	8.8
5	1.7	19.4	105	19.5	029	8.8
	1.5	19.4	105	19.5	029	8.8
	1.4	19.4	105	19.5	029	8.8
	1.3	19.4	105	19.5	029	8.8
6	1.1	21.4	119	19.4	028	8.7
	0.9	21.4	119	19.4	028	8.7
	0.8	21.4	119	19.4	028	8.7
7	0.5	23.9	176	15.9	022	8.6
	0.3	23.9	176	15.9	022	8.6
	0.2	23.9	176	15.9	022	8.6

**Table 5:** Comparison of results of FIS-NC and the developed system.

**Trajectory Number**	$D_{\mathrm{CPA}}\;(\mathrm{nm})$	$T_{\mathrm{CPA}}\;(\min)$	VCD(degree)	$D_{r}\;(\mathrm{nm})$	FIS-NC	Developed System
3	0.644	12.139	0.898	3.803	0.011	0.015
	0.644	11.639	0.917	3.651	0.012	0.016
	0.644	11.139	0.937	3.499	0.015	0.028
4	0.741	10.296	1.182	3.336	0.021	0.031
	0.741	9.796	0.958	3.182	0.028	0.057
	0.741	9.296	0.977	3.028	0.037	0.084
	0.741	8.796	0.996	2.876	0.049	0.119
	0.741	8.296	1.015	2.723	0.058	0.165
	0.741	7.796	1.033	2.572	0.117	0.221
	0.728	7.291	1.083	2.417	0.174	0.283
	0.728	6.791	1.078	2.267	0.231	0.338
	0.728	6.291	1.096	2.118	0.284	0.387
	0.728	5.791	1.114	1.970	0.335	0.429

**Table 6:** Comparison of results of point positioning and timing for collision avoidance.

Division	Guide to Collision Avoidance Rules	FIS-NC	Developed System
$D_{r}$ (nm)	2 to 3	1.970	2.267
$T_{\mathrm{CPA}}$ (min)	6	5.791	6.791

## Data Availability

The data that support the findings of this study are available from the corresponding author upon reasonable request.
